# Identification of chemokine receptors as potential modulators of endocrine resistance in oestrogen receptor–positive breast cancers

**DOI:** 10.1186/s13058-014-0447-1

**Published:** 2014-10-31

**Authors:** Ricardo Ribas, Zara Ghazoui, Qiong Gao, Sunil Pancholi, Aradhana Rani, Anita Dunbier, Mitch Dowsett, Lesley-Ann Martin

**Affiliations:** 1 Breakthrough Breast Cancer Research Centre, Institute of Cancer Research, Fulham Road, London, SW3 6JB UK; 2 Academic Department of Biochemistry, Royal Marsden Hospital, Fulham Road, London, SW3 6JB UK; 3 AstraZeneca, Alderley Park, Macclesfield, SK10 4TG UK; 4 Department of Biochemistry, Otago University, Dunedin, New Zealand

## Abstract

**Introduction:**

Endocrine therapies target oestrogenic stimulation of breast cancer (BC) growth, but resistance remains problematic. Our aims in this study were (1) to identify genes most strongly associated with resistance to endocrine therapy by intersecting global gene transcription data from patients treated presurgically with the aromatase inhibitor anastrazole with those from MCF7 cells adapted to long-term oestrogen deprivation (LTED) (2) to assess the clinical value of selected genes in public clinical data sets and (3) to determine the impact of targeting these genes with novel agents.

**Methods:**

Gene expression and Ki67 data were available from 69 postmenopausal women with oestrogen receptor–positive (ER+) early BC, at baseline and 2 weeks after anastrazole treatment, and from cell lines adapted to LTED. The functional consequences of target genes on proliferation, ER-mediated transcription and downstream cell signalling were assessed.

**Results:**

By intersecting genes predictive of a poor change in Ki67 with those upregulated in LTED cells, we identified 32 genes strongly correlated with poor antiproliferative response that were associated with inflammation and/or immunity. In a panel of LTED cell lines, C-X-C chemokine receptor type 7 (*CXCR7*) and *CXCR4* were upregulated compared to their wild types (wt), and *CXCR7*, but not *CXCR4*, was associated with reduced relapse-free survival in patients with ER+ BC. The CXCR4 small interfering RNA variant (siCXCR4) had no specific effect on the proliferation of wt-SUM44, wt-MCF7 and their LTED derivatives. In contrast, si*CXCR7*, as well as CCX733, a CXCR7 antagonist, specifically suppressed the proliferation of MCF7-LTED cells. si*CXCR7* suppressed proteins associated with G_1_/S transition and inhibited ER transactivation in MCF7-LTED, but not wt-MCF7, by impeding association between ER and proline-, glutamic acid– and leucine-rich protein 1, an ER coactivator.

**Conclusions:**

These data highlight CXCR7 as a potential therapeutic target warranting clinical investigation in endocrine-resistant BC.

**Electronic supplementary material:**

The online version of this article (doi:10.1186/s13058-014-0447-1) contains supplementary material, which is available to authorized users.

## Introduction

Approximately 80% of the breast cancers (BCs) express oestrogen receptor α (ER) at the time of primary diagnosis and depend on oestrogen for their growth and progression. Several endocrine therapies have been developed clinically to target this pathway, including aromatase inhibitors (AIs), which block the conversion of androgens to oestrogens; selective ER modulators such as tamoxifen, which competes with oestrogen for ER; and fulvestrant (ICI 182,780), which binds to ER and targets it for degradation. Despite the efficacy of these agents, many patients eventually relapse with either intrinsic or acquired resistance and, in the majority of cases, continue to express ER.

The elucidation of the molecular pathways governing resistance is crucial for the identification of biomarkers and novel therapeutic strategies. To answer these questions, we, like others, have developed *in vitro* models mimicking relapse on AIs. We have previously demonstrated that crosstalk between ER and type I and type II growth factor receptor tyrosine kinases, most notably ERBB2/EGFR, can circumvent the need for steroid hormones leading to ligand independent activation of the ER or can provide a hypersensitive field in which the ER can respond to very low levels of oestrogen [1]-[8]. Although there is some clinical evidence to support these preclinical findings, only 10% of ER+ tumours coexpress ERBB2, and ERBB2 is rarely overexpressed with acquisition of resistance [9]. This suggests that alternative underlying molecular events remain to be identified.

To address this question, we took a three-way strategy. (1) We examined the intersection of (a) global gene transcription data from ER+ breast tumours of patients treated with neoadjuvant anastrazole with (b) data from MCF7 cells adapted to long-term oestrogen deprivation (LTED). (2) We assessed the clinical value of selected genes in public clinical data sets. (3) We determine the function and utility of these proteins as novel therapeutic targets.

**Figure 1:**
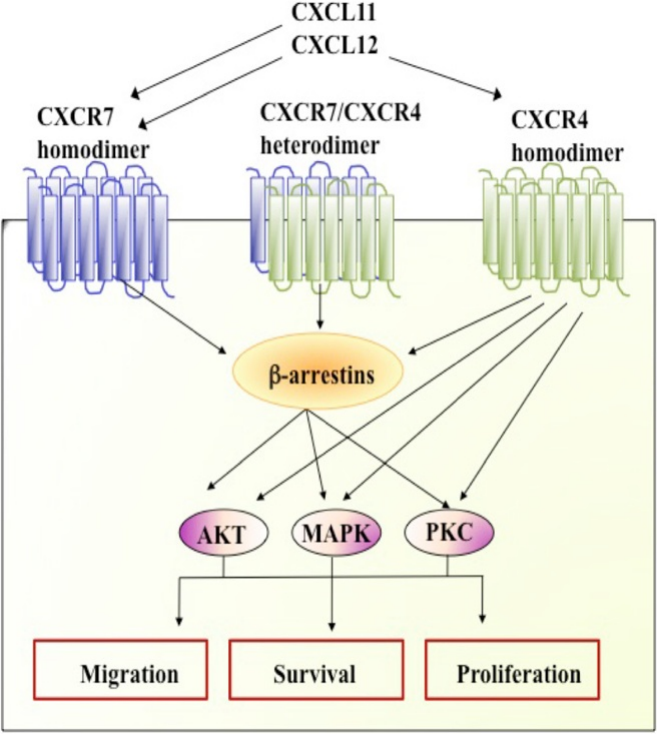
**Schematic representation of signalling pathways of C-X-C chemokine receptors CXCR4 and CXCR7.** MAPK, Mitogen-activated protein kinase; PKC, Protein kinase C.

In particular, we found genes associated with inflammation and immunity, such as C-X-C chemokine receptors (CXCRs), as potential biomarkers of poor response. CXCRs belong to the family of seven transmembrane receptors responsible for the initiation of a cascade of signal transduction events (Figure 1). Previous studies have shown that CXCRs are involved in the development of several types of cancer by promoting cell growth, metastasis and resistance to chemotherapy [10].

In the present study, we provide evidence for the role of CXCR7 in endocrine resistance. Clinical data demonstrate that CXCR7 is associated with poor relapse-free survival (RFS) in BC patients. Additionally, *in vitro* models of endocrine resistance provide evidence of novel crosstalk between ER, PELP/MNAR (proline-, glutamic acid- and leucine-rich protein 1/modulator of nongenomic activity of ER) and CXCR7, highlighting this axis as a potential therapeutic target in endocrine-resistant BC.

## Methods

### Cell culture

Human BC cell lines were obtained from the American Type Culture Collection (Manassas, VA, USA). MCF7, HCC1428, ZR75.1, T47D and SUM44 (ER+) cell lines were cultured in phenol red–free RPMI 1640 medium supplemented with 10% dextran-coated charcoal (DCC)–stripped serum and 1 nM oestradiol (E2). LTED derivatives modelling resistance to an AI were cultured in the absence of E2. ER- MDA-MB-231 cells were cultured in RPMI 1640 medium containing 10% foetal bovine serum.

### Gene expression microarray analysis

#### Cell lines

Global gene expression data were available from MCF7 cells adapted to LTED [11] (ArrayExpress accession number E-MTAB-922). Differential gene expression analyses between both cell models were performed in a pairwise fashion using BRB-ArrayTools (developed by Dr. Richard Simon and the BRB-ArrayTools Development Team).

#### Breast tumours

Paired core-cut tumour biopsies were obtained from 69 postmenopausal women with stages I to IIIB ER+ early BC. The biopsies were obtained at baseline and after a 2-week course of single-agent neoadjuvant anastrozole therapy. RNA extracted from biopsies was hybridised onto HumanWG-6 v2 Expression BeadChips (Illumina, San Diego, CA, USA). Global gene expression and Ki67 data were available [12]. Spearman's rank correlation coefficient was used to identify the genes that were predictive of good or poor change in Ki67 between baseline gene expression and Ki67 protein level change. Data were extracted, transformed, normalised and filtered using the same methods as those used for the cell lines [11]. The detailed methodology used for this work, as well as the patient demographics, are published elsewhere [12]. This study received approval from an institutional review boards at each site and was conducted in accordance with the 1964 Declaration of Helsinki and International Conference on Harmonization/Good Clinical Practice guidelines. Written informed consent was obtained from each patient before participation.

### Analysis of clinical public data sets

Survival analysis was carried out using the publicly available online tool KM plotter [13]. Prognostic values for CXCR4 and CXCR7 were evaluated using data from 500 untreated ER+ patients (determined by immunohistochemistry) with follow-up of 12 years. The highest quartile of the gene expression was used to dichotomise the patient population into high and low groups. The same parameters were used to determine the on-treatment effect of tamoxifen in 840 patients with follow-up data at 5 and 12 years. A significant impact on RFS was assumed to be present if the log-rank *P*-value was <0.05.

### Proliferation assays

Wild-type MCF7 (wt-MCF7) and MCF7-LTED cells were seeded into 10% DCC medium in 96-well plates at 3 × 10^3^ and 1.5 × 10^3^ cells/well, respectively. wt-SUM44 and SUM44-LTED cells were seeded at a density of 2× 10^3^ cells/well. Twenty-four hours later, monolayers were transfected with 50 nM of ON-TARGET*plus* siRNA (Dharmacon/GE Healthcare Life Sciences, Lafayette, CO, USA) against nontargeting pool (si*control*), human CXCR4 pool, human CXCR7 pool or deconvoluted small interfering RNAs (siRNAs) using Lipofectamine RNAiMax reagent (Invitrogen, Carlsbad, CA, USA) for 24 hours. Cells were subsequently treated with vehicle (either DCC alone or DCC with 1 nM E2) for 4 days. For experiments involving inhibitors, cells were cultured in DCC for 3 days and seeded as specified above. Twenty-four hours later, cells were treated with the CXCR7 antagonist CCX733 or with CCX704, an analogue with no binding affinity to CXCR7 [14] (ChemoCentryx, Mountain View, CA, USA). Drugs were diluted in dimethyl sulphoxide, and monolayers were treated at concentrations between 0 and 1,000 nM. Effects at concentrations above 5,000 nM were considered nonspecific [15]. Cells were treated for 6 days, with a treatment change on day 3. Cell viability was determined using the CellTiter-Glo Luminescent Cell Viability Assay (Promega, Madison, WI, USA). Each experiment was performed at least three times. A control 24-well plate was harvested 48 hours after siRNA transfection to verify the knockdown of CXCR7 by quantitative RT-PCR (qRT-PCR).

### Fluorescence-activated cell sorting analysis

Cell surface expression of CXCR7 was determined using fluorescence-activated cell sorting (FACS). MDA-MB-231, wt-MCF7 and MCF7-LTED cells were cultured under their respective basal conditions. Cells were collected and incubated with mouse monoclonal CXCR7 antibody (11G8; a gift from Mark Penfold, ChemoCentryx) or mouse immunoglobulin G1 (IgG1; Dako, Carpinteria, CA, USA) for 30 minutes at 4°C. After a series of washes, cells were incubated with Alexa Fluor 488 goat anti-mouse IgG antibody and counterstained with 4-,6-diamidino-2-phenylindole (DAPI). CXCR7 content was determined in a BD LSR II flow cytometer using BD FACSDiva software (BD Biosciences, San Jose, CA, USA), and the data were analysed using the FlowJo software program (FlowJo, Ashland, OR, USA).

### Apoptosis assay

Apoptosis was measured using the Cell Death Detection ELISA^PLUS^ kit (Roche Life Science, Indianapolis, IN, USA) according to the manufacturer's instructions.

### Cell cycle analysis

Cells were transfected with si*control* or si*CXCR7* as described above and subsequently treated for 48 hours with DCC containing vehicle or 1 nM E2. Cells were collected and fixed overnight with 90% ethanol, then stained with bromodeoxyuridine and propidium iodide (Sigma-Aldrich, St Louis, MO, USA) and analysed as previously described [16].

### Transcriptional assays

Cells were plated and transfected with si*control* or si*CXCR7* and were treated the following day with oestrogen response element (ERE)–linked luciferase reporter and β-galactosidase constructs [11]. The activity of luciferase (Promega) and β-galactosidase (GalactoStar; Applied Biosystems, Foster City, CA, USA) was measured using a luminometer. Each experiment was performed three times. A control plate was used to verify the knockdown of CXCR7 at the RNA level.

### Real-time quantitative PCR

mRNA was extracted using the RNeasy Mini Kit (QIAGEN, Valencia, CA, USA), and quantification was performed using Agilent 2100 Bioanalyzer Expert Software (version B.02.03) with RNA Nano LabChip kits (Agilent Technologies, Wokingham, UK). Total RNA was reverse-transcribed using SuperScript III reverse transcriptase (Invitrogen) and random primers in accordance with the manufacturer's instructions. cDNA was subjected to qRT-PCR experiments in triplicate. TaqMan gene expression assays (Applied Biosystems) were performed to quantify *CXCR4* (Hs00237052_m1), *CXCR7* (Hs00604567_m1), *CXCL11* (Hs00171138_m1), *CXCL12* (Hs00171022_m1), *TFF1* (Hs00907239_m1) and *BCL2* (Hs00608023_m1), together with *FKBP15* (Hs00391480_m1) as a housekeeping gene, to normalise the data. To measure gene expression, we used the ΔΔCt method for relative quantification or a standard curve generated from serial dilutions of reference cDNA from pooled BC cell lines for absolute quantification.

### Immunohistochemistry

Formalin-fixed, paraffin-embedded cells were stained for CXCR7 (1:2,000 dilution with antibody 11G8 for detection) using the EnVision FLEX + High pH kit according to the manufacturer's instructions (Dako), and the procedure was conducted using the Autostainer Immunostaining System (Dako).

### Western blot analysis

Whole-cell extracts were generated as described previously [5]. Equal amounts of protein were resolved by SDS-PAGE and transferred to nitrocellulose membranes (Whatman/GE Healthcare Life Sciences, Maidstone, UK). Antigen–antibody interactions were detected with Amersham ECL detection reagents (Amersham/GE Healthcare Life Sciences, Little Chalfont, UK). The following primary antibodies were used: phosphorylated AKT (pAKT), cyclin D1, phosphorylated Rb807, CDK4, CDK7, Bcl-2, phosphorylated p38, phosphorylated protein kinase A (pPKA), phosphorylated stress-activated protein kinase c-Jun N-terminal kinase (SAPK/pJNK) and phosphorylated ER serine 118 and serine 167 (all from Cell Signaling Technology, Danvers, MA, USA); total ER and poly(ADP-ribose) polymerase (PARP) (Santa Cruz Biotechnology, Santa Cruz, CA, USA); phosphorylated extracellular signal-regulated kinases 1 and 2 (pERK1/2), α-tubulin and β-actin (Sigma-Aldrich); BUB1 and pCDK7 (Abcam, Cambridge, UK); cyclin B1 (Thermo Scientific, Rockford, IL, USA); and β-arrestin 1 and β-arrestin 2 (gift from Jeffrey Benovic). Secondary antibodies (anti-mouse and anti-rabbit horseradish peroxidase) were obtained from Dako.

### Immunofluorescence and confocal studies

Cells were seeded onto glass coverslips in 10% DCC media. Nonsense or siRNA against human CXCR7 was transfected at 50 nM using Lipofectamine RNAiMax. After 24 hours, cells were treated with DCC media for 48 hours. Monolayers were fixed in 4% paraformaldehyde in phosphate-buffered saline (PBS) for 15 minutes, then permeabilised with 0.5% Triton X-100 in PBS for 10 minutes. Cells were subsequently incubated with the anti-CXCR7 antibody 11G8 for 2 hours. Coverslips were incubated in Alexa Fluor 488–labelled secondary antibody (1:1,000; Molecular Probes, Eugene, OR, USA) for 1 hour. Nuclei were counterstained with DAPI (1:10,000; Invitrogen). Coverslips were mounted onto glass slides using VECTASHIELD Mounting Medium (Vector Laboratories, Peterborough, UK). Images were collected sequentially in the two channels on an LSM 710 confocal microscope (Zeiss, Oberkochen, Germany).

### Immunoprecipitation

Cell lysates were precleared, incubated with primary antibodies (HC-20 antibody for ER, Santa Cruz Biotechnology; PELP1 antibody, Bethyl Laboratories, Montgomery, TX, USA) at 4°C overnight. Immunocomplexes were recovered using protein G, washed six times in extraction buffer and resolved by SDS-PAGE, as specified above.

### Statistical analysis

Statistical analysis was performed using Student's *t*-test for the qRT-PCR experiments, cycle analyses, and proliferation, apoptosis and transcription assays.

## Results

### C-X-C chemokine receptors as potential molecular markers associated with resistance to aromatase inhibitors

**Figure 2:**
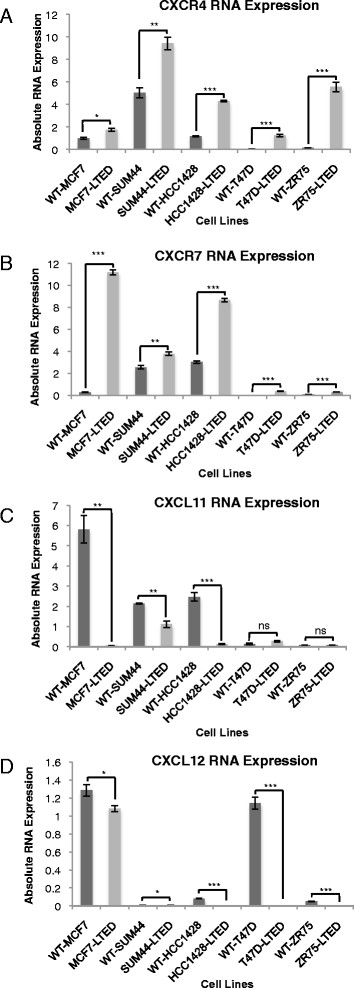
***CXCR4*** **,** ***CXCR7, CXCL11*** **and** ***CXCL12*** **expression in five oestrogen receptor–positive human breast cancer cell lines adapted to long-term oestrogen deprivation.** Expression of *CXCR4* **(A)**, *CXCR7* **(B)**, *CXCL11* **(C)** and *CXCL12* **(D)** in wild-type (WT) and their corresponding long-term oestrogen-deprived (LTED) cell lines. CXCR, C-X-C chemokine receptor; CXCL, C-X-C chemokine ligand. Bars represent – standard error of the mean (SEM) *P<0.05 ** P<0.01, ***P<0.001

Global gene expression and Ki67 data were available from paired baseline and 2-week posttreatment core-cut tumour biopsies obtained from 69 postmenopausal women with stages I to IIIB ER+ early BC who received single-agent neoadjuvant anastrozole. These data have been reported in detail elsewhere [12]. In BC, immunohistochemical assessment of Ki67 has been validated as a dynamic biomarker of endocrine treatment efficacy in samples taken before, during and after neoadjuvant endocrine therapy [17]. To identify clinically relevant molecular markers associated with resistance to AIs that may be assessed in our *in vitro* models, we identified the intersection between genes that were significantly upregulated in MCF7 cells adapted to LTED [11] and genes predictive of a poor change in Ki67 in the clinical samples. Using *P* <0.01 for clinical data and *P* <0.001 as the levels of significance for cell line data, 32 genes were identified as present in both data sets. Most notably, genes associated with inflammation and immunity, such as CXCRs, were evident (Table 1 and Additional file 1: Figure S1). This was of particular interest because previously we had shown by molecular profiling of AI-treated postmenopausal breast tumours that pretreatment expression of an inflammatory signature correlated with resistance to therapy [12]. Analysis of changes in gene expression in LTED cells versus the wt-MCF7 cells showed that *CXCR4* was upregulated 1.5-fold (*P* = 0.0005) and CXCR7, the suggested coreceptor for CXCR4 [18], was the top upregulated gene, with an increase of 17.9-fold (*P* = 3.6 × 10^-6^) compared to wt-MCF7 cells. Crosstalk between CXCR4 and CXCR7 is well established [19] and is known to regulate more than 170 common genes (data not shown). Therefore, we sought to determine their expression and that of their associated ligands (*CXCL11* and *CXCL12*) in a panel of LTED cells. *CXCR4* and *CXCR7* were significantly increased in all LTED cell line derivatives compared to their associated parental cells. *CXCL11* expression was restricted to wt-MCF7, wt-SUM44 and wt-HCC1428 cells and was significantly reduced in their LTED derivatives. *CXCL12* was undetected in wt-SUM44 and SUM44-LTED cells, and it was lost in all other LTED cells except MCF7-LTED (Figure 2).

**Table 1:** **Identification of 32 genes associated with poor response to aromatase inhibitor therapy**

Gene	Name	***In vitro***		Clinical	
		Fold change	***P*** -value	Correlation coefficient	***P*** -value
*ACTR2*	ARP2 actin-related 2 homolog (yeast)	1.82	2.74E-05	-0.378	9.68E-04
*AP1B1*	Adaptor-related protein complex 1, β_1_ subunit	1.67	6.25E-04	-0.398	4.96E-04
*ARID3A*	AT rich interactive domain 3A (BRIGHT-like)	1.60	1.36E-04	-0.352	2.25E-03
*CCM2*	Cerebral cavernous malformation 2	1.94	5.07E-05	-0.36	1.72E-03
***CXCR4***	**Chemokine (C-X-C motif) receptor 4**	1.51	5.05E-04	-0.302	9.26E-03
*DTX2*	Deltex homolog 2 (*Drosophila*)	2.24	8.19E-04	-0.338	3.41E-03
*EML4*	Echinoderm microtubule–associated protein–like 4	2.50	1.60E-06	-0.336	3.59E-03
*FAM38A*	Family with sequence similarity 38, member A	3.59	5.02E-05	-0.338	3.42E-03
*FBXL10*	Lysine (K)-specific demethylase 2B	3.12	8.80E-06	-0.3	9.70E-03
*IFNAR2*	Interferon (α, β and ω) receptor 2	1.50	2.36E-04	-0.313	6.79E-03
*IFNGR1*	Interferon γ receptor 1	1.85	1.02E-04	-0.356	1.99E-03
*IGF2R*	Insulin-like growth factor 2 receptor	2.93	1.20E-06	-0.319	5.84E-03
*KIAA0513*	KIAA0513	4.21	2.42E-04	-0.379	9.43E-04
*LILRB1*	Leukocyte immunoglobulin-like receptor, subfamily B, member 1	1.61	1.77E-04	-0.389	6.80E-04
*LILRB3*	Leukocyte immunoglobulin-like receptor, subfamily B, member 3	2.06	2.50E-06	-0.366	1.42E-03
*MAP3K7*	Mitogen-activated protein kinase 7	1.97	7.90E-06	-0.334	3.84E-03
*MFHAS1*	Malignant fibrous histiocytoma-amplified sequence 1	2.11	1.79E-05	-0.299	9.76E-03
*MGAT1*	Mannosyl glycoprotein acetylglucosaminyltransferase	1.70	1.33E-04	-0.313	6.78E-03
*MICAL1*	Microtubule-associated monooxygenase, calponin/LIM domain	2.43	7.00E-07	-0.385	7.65E-04
*NPC2*	Niemann-Pick disease, type C2	2.37	3.09E-05	-0.421	2.12E-04
*PHACTR2*	Phosphatase and actin regulator 2	3.20	1.22E-05	-0.35	2.39E-03
*PLEKHF1*	Pleckstrin homology domain containing, family F, member 1	2.13	3.51E-05	-0.369	1.31E-03
*RGS19*	Regular of G-protein signaling 19	1.52	7.14E-04	-0.474	2.54E-05
*SLC15A4*	Solute carrier family 15, member 4	1.54	9.13E-05	-0.307	7.91E-03
*ST8SIA4*	ST8 α-*N*-acetyl-neuraminide α-2,8-sialyltransferase 4	2.45	2.74E-04	-0.32	5.64E-03
*TNRC6B*	Trinucleotide repeat containing 6B	2.13	1.90E-04	-0.3	9.74E-03
*TTYH3*	Tweety homolog 3 (*Drosophila*)	2.72	4.65E-04	-0.408	3.42E-04
*WDFY1*	WD repeat and FYVE domain containing 1	2.27	3.00E-06	-0.349	2.40E-03
*XPO6*	Exportin 6	1.80	4.64E-04	-0.312	6.99E-03
*YWHAH*	Tyrosine/tryptophan monooxygenase activation protein	1.44	4.83E-04	-0.365	1.50E-03
*ZBED4*	Zinc finger, BED-type containing 4	1.59	5.93E-05	-0.337	3.48E-03
*ZCCHC11*	Zinc finger, CCHC domain containing 11	1.36	4.75E-04	-0.433	1.36E-04

### Effect of CXCR4 and CXCR7 downregulation upon cell proliferation

**Figure 3:**
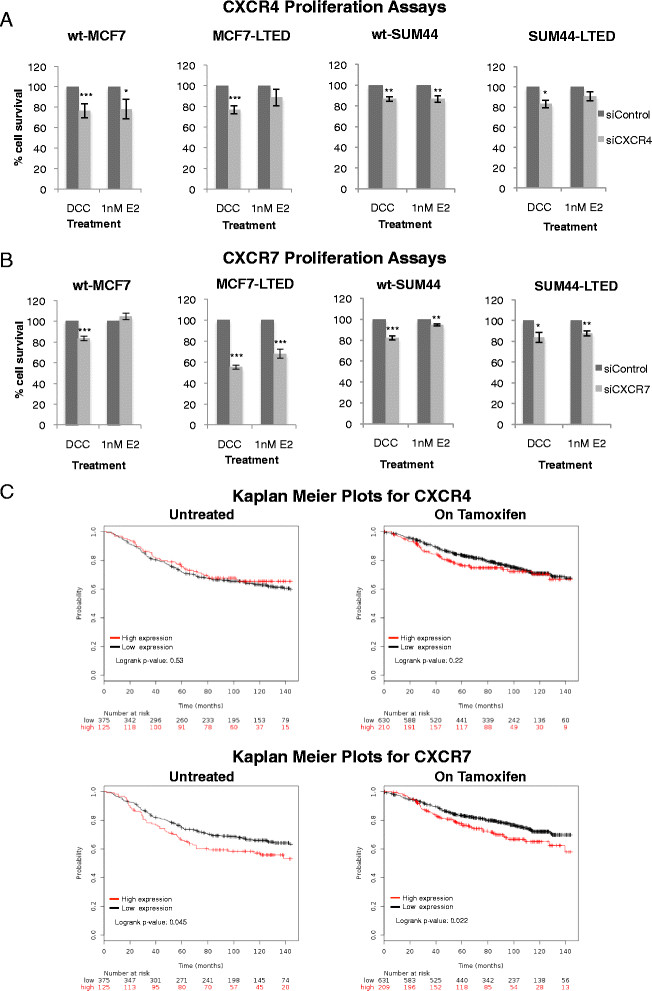
**Analysis of CXCR4 and CXCR7 in cell lines and clinical data on association with recurrence of oestrogen receptor–positive breast cancer.** wt-MCF7, MCF7-LTED, wt-SUM44 and SUM44-LTED cells were transfected with si*CXCR4* **(A)** or si*CXCR7* **(B)** versus si*control* × exogenous oestradiol (E2) (1 nM). Cells were cultured for 6 days. Cell survival was measured using CellTiter-Glo. The data are expressed as fold changes relative to si*control*. Each treatment was carried out with eight replicates. The data shown are representative of a minimum of five independent experiments. Bars represent × standard error of the mean (SEM). *P <0.05, **P <0.01, ***P <0.001. **(C)** Kaplan-Meier analysis of the influence of *CXCR4* and *CXCR7* on relapse-free survival (RFS) in patients with oestrogen receptor–positive (ER+) breast cancer (BC), who were either treatment-na–ve or treated with tamoxifen, whose data were derived from publicly available clinical BC data sets collected over 12 years [13]. Data were stratified by the highest quartile versus the rest. CXCR, Chemokine C-X-C receptor; DCC, Dextran-coated charcoal; LTED, Long-term oestrogen deprivation; si, Small interfering; wt, Wild type.

To determine the relevance of CXCR4 and CXCR7 on cell proliferation, we selected LTED cell lines that retained expression of ER (Additional file 2: Figure S2) and at least one of the ligands for CXCR4 and CXCR7 (Figure 2), along with their parental lines. si*CXCR4* showed a minimal, nonspecific antiproliferative effect in the four cell lines tested with or without E2 (Figure 3A). In contrast, compared to si*control*, si*CXCR7* significantly inhibited proliferation of MCF7-LTED cells by 45% (*P* <0.001) in the absence of E2 and by 32% (*P* <0.001) in the presence of E2. Depletion of CXCR7 in wt-MCF7 had a statistically significant but quantitatively modest effect, suppressing proliferation by 17% in the absence of E2 (*P* <0.05) and no significant effect in the presence of E2 (Figure 3B). In contrast, suppression of CXCR7 showed no selective inhibition of proliferation in wt-SUM44 and SUM44-LTED. As CXCR4 and CXCR7 show a high degree of crosstalk, we assessed whether the combination of si*CXCR4* and si*CXCR7* would induce a greater antiproliferative effect compared with si*CXCR7* alone. No significant effect of the combination was noted in any of the cell lines tested, with the exception of the wt-MCF7 in the presence of E2, which showed a modest alteration that just barely met statistically significance (Additional file 3: Figure S3). These data suggest that the antiproliferative effect of si*CXCR7* in MCF7-LTED cells maybe context-specific.

### Relationship between CXCR4 and CXCR7 expression and long-term outcome in oestrogen receptor–positive breast cancer patients

Using public data from untreated patients with ER+ BC with 12 years of follow-up, high levels of *CXCR7* were found to be associated with poor RFS. In contrast, *CXCR4* was not significantly associated with RFS (Figure 3C). Using gene expression data from patients treated with tamoxifen [13], *CXCR7* was found to be associated with a significant reduction in time to relapse (0 to 12 years), whereas *CXCR4* was not (Figure 3C). Assessment of *CXCR4* expression over the course of 0 to 5 years showed a significant association with poor RFS during this time frame (Additional file 4: Figure S4A), whereas the RFS curves closed after 5 years, indicating little association with late recurrence after treatment had ceased (Figure 3C).

To model this clinical observation *in vitro*, wt-MCF7 cells were deprived of E2 for 1 week (short-term oestrogen deprivation, STED) or for 20 weeks (LTED). Alterations in *CXCR4* and *CXCR7* expression were evaluated by qRT-PCR. After 1 week (STED), both *CXCR4* and *CXCR7* increased significantly (6-fold and 13-fold, respectively) compared with the parental cells. However, after 20 weeks of deprivation, *CXCR4* was significantly reduced in comparison with the STED (1.7-fold decreased, *P* <0.001). In contrast, *CXCR7* was significantly increased (4-fold, *P* = 0.01) (Additional file 4: Figure S4B).

In summary, although CXCR4 was minimally upregulated in the LTED cells, a model of late relapse, it may no longer play a significant role in proliferation, as indicated by our siRNA knockdown studies. In contrast, CXCR7 was highly expressed and had specific targetable proliferative activity in this setting.

On the basis of these clinical and *in vitro* data, we focused our attention on the role of CXCR7 in late relapse.

### Characterisation of CXCR7 in long-term oestrogen-deprived MCF7 versus wild-type MCF7

Using deconvoluted siRNAs targeting *CXCR7*, we confirmed the antiproliferative effect of CXCR7 depletion in MCF7-LTED cells (Additional file 5: Figure S5). Controversy has been raised regarding the use of CXCR7 antibodies for immunoblotting [20]; therefore, we performed immunohistochemical and FACS analysis to compare the protein expression of CXCR7 between wt-MCF7 and MCF7-LTED, having first confirmed antibody specificity using confocal microscopy, which showed loss of staining after si*CXCR7*. There was an increase of CXCR7 protein levels in MCF7-LTED compared to wt-MCF7 cells. MDA-MB-231 cells, which do not express CXCR7, were used as a negative control for both MCF7-LTED and wt-MCF7 cells by immunohistochemical and FACS analysis (Additional file 6: Figure S6).

### CXCRdepletion does not enhance apoptosis in vitro

**Figure 4:**
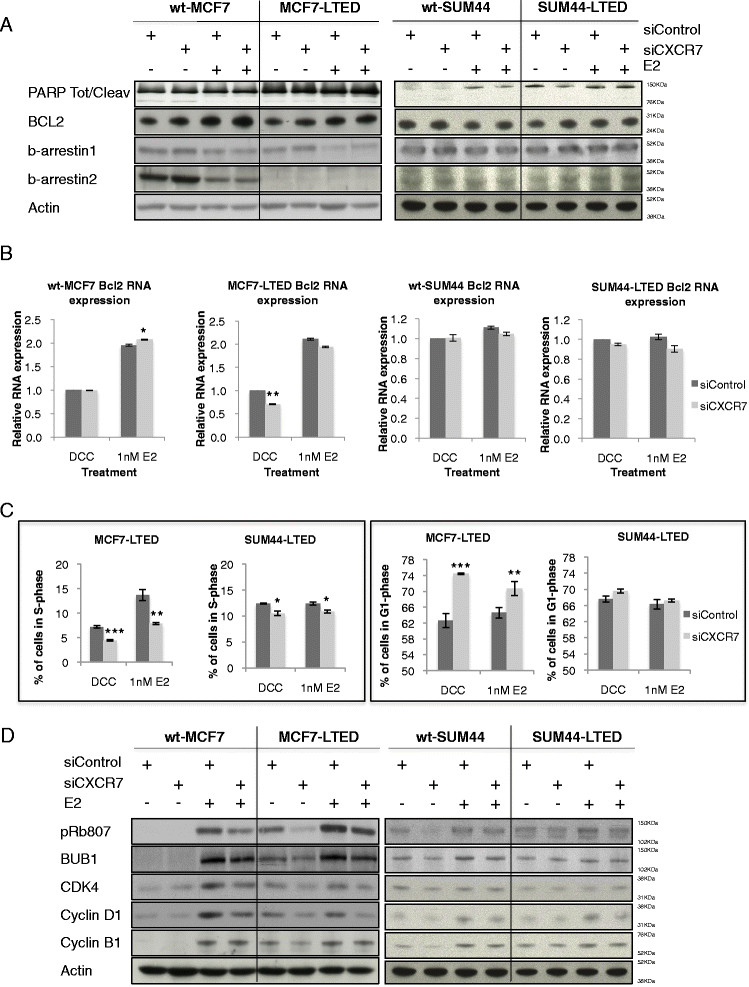
**Depletion of CXCR7 causes cell cycle arrest, but not apoptosis.** wt-MCF7, MCF7-LTED, wt-SUM44 and SUM44-LTED cells were transfected with si*control* or si*CXCR7* × oestradiol (E2) for 72-hours. **(A)** Whole-cell extracts were immunoblotted and assessed for changes in expression of BCL2, cleaved poly(ADP-ribose) polymerase (PARP) and β-arrestins 1 and 2. **(B)** Changes in *BCL2* were assessed by quantitative RT-PCR. Data are expressed relative to dextran-coated charcoal (DCC)–treated si*control*. **(C)** Assessment of alteration in S- and G_1_-phase accumulation as a result of CXCR7 depletion. Data are expressed as mean × SEM. *P <0.05, **P <0.01, ***P <0.001. **(D)** Immunoblot analysis of cell cycle markers in response to CXCR7 depletion. Data shown are representative of a minimum of three independent experiments. CXCR, C-X-C chemokine receptor; LTED, Long-term oestrogen deprivation; si, Small interfering; wt, Wild type.

CXCR7 can signal through β-arrestin [21]. β-arrestin has been linked to prosurvival signalling by enhancing *BCL2* expression by promoting acetylation of histone H4 at the *BCL2* promoter [22]. Therefore, we hypothesised that increased CXCR7 might lead to increased expression of BCL2 via β-arrestin. si*CXCR7* significantly reduced *BCL2* transcript levels in MCF7-LTED, but not in SUM44-LTED cells. No effect upon BCL2 protein expression was evident in either cell line model. Furthermore, depletion of CXCR7 did not affect PARP cleavage (Figures 4A and 4B), and no increase in cell death was evident using live/dead viability assays. Similarly, no increase was found in the sub-G_1_ fraction in response to si*CXCR7* (Additional file 7: Figure S7). si*CXCR7* had no effect on expression of β-arrestin 1 or 2 in either cell line. Most notably, no significant alteration in the expression of β-arrestin 1 was evident between the cell lines, although MCF7-LTED cells showed loss of expression of β-arrestin 2. Furthermore, both β-arrestins 1 and 2 were suppressed by addition of exogenous E2 in the MCF7-LTED and its wild type, although this was not the case in the wt-SUM44 and SUM44-LTED cells (Figure 4A). Therefore, these data suggest that crosstalk between CXCR7 and β-arrestin did not impede apoptosis in the LTED cell line.

### CXCR7 depletion blocks cell cycle progression and G1/S transition

We performed flow cytometry to assess whether the antiproliferative effect of si*CXCR7* in MCF7-LTED and SUM-44 cells results from cell cycle arrest in response to si*CXCR7*. si*CXCR7* caused a significant decrease in S-phase without and with E2 (40% decrease without E2 (p = 0.0007) and 42% decrease with E2 (*P* = 0.006)), and a concomitant increase in G_1_-phase, compared to si*control* in MCF7-LTED cells. In contrast, si*CXCR7* caused a minimal decrease in the number of cells in S-phase in the SUM44-LTED cells (15% (*P* = 0.01) in DCC and 12% (*P* = 0.03) in E2) (Figure 4C). This is in keeping with the limited antiproliferative effect noted previously (Figure 3A). Furthermore, si*CXCR7* had negligible effects on expression of cell cycle regulatory proteins in either wt-SUM44 or SUM44-LTED cells. In contrast, si*CXCR7* decreased expression of cell cycle regulatory proteins, predominantly in the MCF7-LTED cells in the absence of E2 but also in the presence of E2. No suppression of these already low levels of cell cycle proteins in response to si*CXCR7* was observed in wt-MCF7 cells in the absence of E2, and only a minimal effect was evident in the presence of E2 (Figure 4D).

### CXCR7 knockdown affects oestrogen receptor-mediated transactivation in MCF7-LTED cells

**Figure 5:**
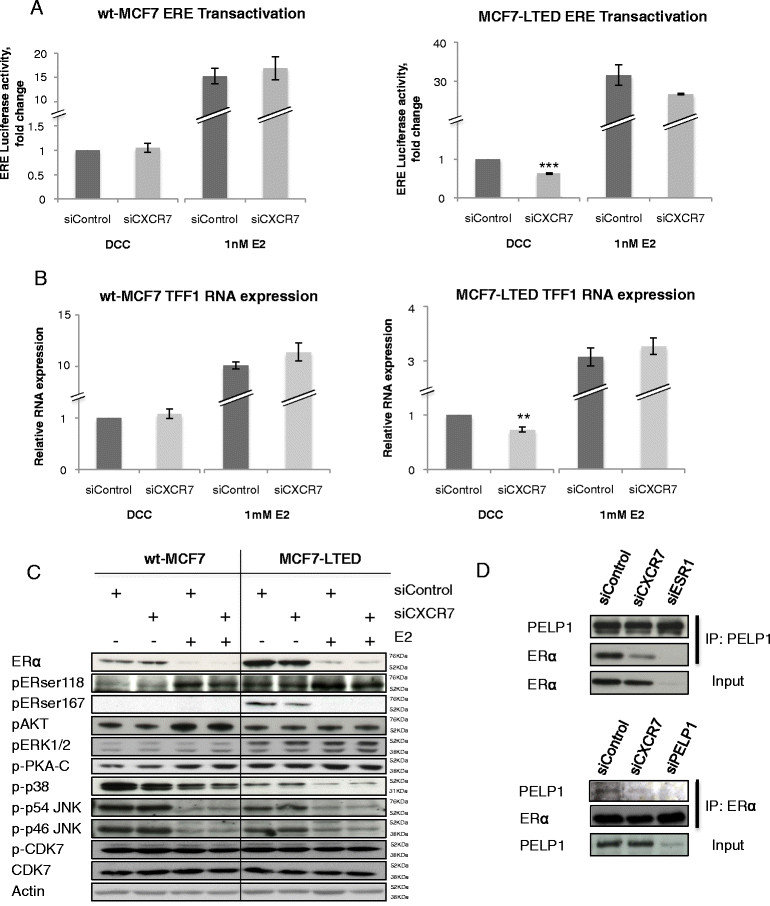
**CXCR7 is required for the interaction between oestrogen receptor and PELP1.** wt-MCF7 and MCF7-LTED cells were transfected with si*control* or si*CXCR7*. **(A)** Oestrogen receptor/oestrogen response element (ER/ERE) transactivation was monitored with an ERE-linked luciferase reporter and pCH110 (β-galactosidase control) and expressed relative to dextran-coated charcoal (DCC) control. **(B)** Expression of *TFF1* was assessed by quantitative RT-PCR, as previously described. Error bars represent × SEM. *P <0.05, **P <0.01. **(C)** Immunoblot analysis of kinases associated with phosphorylation of ER in response to CXCR7 depletion. **(D)** MCF7-LTED cells were left untreated or transfected with si*control*, si*CXCR7*, si*PELP1* or si*ESR1*, immunoprecipitated for PELP1; and immunoblotted for ER or vice versa. The data shown are representative of three independent experiments. CXCR, Chemokine C-X-C receptor; E2, Oestradiol; IP, Immunoprecipitation; JNK, c-Jun N-terminal kinase; LTED, Long-term oestrogen deprivation; PELP, Proline-, glutamic acid- and leucine-rich protein 1; si, Small interfering; wt, Wild type.

MCF7-LTED cells upregulate ER expression and are dependent on ER/ERE-driven transcription for proliferation [5]. si*CXCR7* had no effect on ER-mediated transcription in the wt-MCF7 cells in the presence or absence of E2. However, depletion of CXCR7 resulted in a 40% decrease (*P* <0.001) in the MCF7-LTED cells compared to si*control* in the absence of E2. No significant changes were observed in MCF7-LTED cells in the presence of E2 (Figure 5A). Similarly, si*CXCR7* had no effect on the expression of *TFF1*, an endogenous oestrogen-regulated gene in wt-MCF7 cells, but resulted in a 30% reduction (*P* <0.01) in the MCF7-LTED cells in the absence of E2. si*CXCR7* knockdown did not affect *TFF1* expression in MCF7-LTED cells in the presence of E2 (Figure 5B).

### Depletion of CXCRdoes not affect downstream pathways associated with oestrogen receptor phosphorylation

Studies have suggested that aberrant kinase activity can lead to the ligand-independent phosphorylation of ER at serine 167 and serine 118. si*CXCR7* in the presence or absence of E2 had no effect on expression of pERK1/2, pAKT, p38, pCDK7, pPKA or pJNK, all of which are phosphorylated kinases associated with ER function. Furthermore, no significant changes in the phosphorylation of ER were evident (Figure 5C).

### CXCR7 depletion impeded the interaction between oestrogen receptor and coactivator PELP1

ER function is dependent on the recruitment of coactivators. Recent studies have suggested that CDK4 is required for phosphorylation of PELP1, allowing interaction with ER [23]. We previously showed that si*CXCR7* significantly reduced expression of CDK4 in MCF7-LTED cells (Figure 4D); therefore, we postulated that si*CXCR7* may impede the interaction between ER and PELP1, leading to a reduction in ER-mediated transcription and concomitant decrease in proliferation. si*CXCR7* caused a significant reduction in the association between PELP1 and ER in LTED cells compared to si*control*. Furthermore si*ESR1* and si*PELP1* produced similar results (Figure 5D).

### CCX733 specifically inhibits proliferation of MCF7-LTED cells, but not wt-MCF7 cells

**Figure 6:**
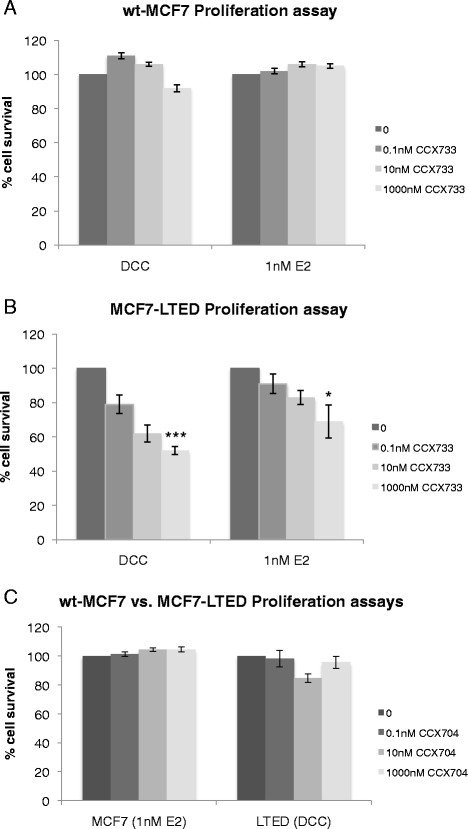
**Treatment of wt-MCF7 and MCF7-LTED cells with escalating concentrations of CXCR7 inhibitor CCX733 with or without exogenous oestradiol.** wt-MCF7 cells **(A)** and MCF7-LTED cells **(B)** were treated with escalating concentrations of CCX733 for 6 days. Cell survival was measured using CellTiter-Glo. The data are expressed as fold changes relative to vehicle-treated control (0). **(C)** MCF7-LTED and wt-MCF7 cells were treated with CCX704 as a negative control. CXCR, Chemokine C-X-C receptor; DCC, Dextran-coated charcoal; E2, oestradiol; LTED, Long-term oestrogen deprivation; wt, Wild type. *P<0.05 ** P<0.01, ***P<0.001.

To address whether CXCR7 could be a clinically relevant drug target, we assessed the sensitivity of MCF7-LTED versus wt-MCF7 cells to escalating concentrations of CCX733, a specific antagonist of the activity of CXCR7, but not of CXCR4 activity [24]. CCX733 had no effect on the proliferation of wt-MCF7 cells with or without E2 (Figure 6A), but caused a dose-dependent decrease in proliferation of MCF7-LTED cells, which was enhanced in the absence of E2 (Figure 6B), consistent with our previous observation using si*CXCR7* (Figure 3B). As a negative control, we assessed the effect of CCX704, an analogue with no binding affinity for CXCR7. CCX704 had no antiproliferative effect in either cell line, as expected (Figure 6C). To further address the combination effects, we combined CXCR7 depletion with fulvestrant, which showed a 35% enhancement in the antiproliferative effect of the combination compared with fulvestrant alone (Additional file 8: Figure S8).

## Discussion

To identify genes associated with resistance to AI therapy that could be manipulated in our model system, we identified the overlap between genes whose expression changes in MCF7 cells adapted to LTED and genes whose expression relates to the antiproliferative response of ER+ tumours from patients treated with neoadjuvant anastrazole. Using this strategy, we removed any contribution from the tumour stroma, thus allowing identification of pathways that were solely tumour-associated. The predominant genes linked with poor response to anastrazole therapy were those associated with inflammation and immunity, such as CXCRs. *In vitro* analysis of a panel of cell lines modelling adaptation to LTED showed that both *CXCR4* and *CXCR7* were overexpressed compared to the parental cell lines. CXCR4, CXCR7 and their shared ligand CXCL12 are thought to affect several major signalling pathways involved in cell survival, proliferation and metastasis [10]. High expression of CXCR4 has been associated with invasion and migration of tumour cells in patients with BC [25],[26], a role confirmed both *in vivo* and *in vitro* [27],[29].

To confirm the clinical relevance of CXCR4 and CXCR7, we assessed the effect of expression in publicly available clinical data sets, restricting significance only to patients with ER+ disease who had not received adjuvant endocrine treatment. Notably, *CXCR7* expression, but not *CXCR4* expression, was prognostic for poor RFS. Furthermore, interrogation of global gene expression data from a cohort of ER+ patients who had received adjuvant tamoxifen therapy showed similarly that CXCR7, but not CXCR4, was associated with poor RFS. Further analysis suggested that CXCR4 was in fact associated with early (0 to 5 years) but not late relapse.

As the large majority of patients at relapse continue to express the ER, we postulated that the role of *CXCR7* in resistance to AI therapy was associated with continued expression of ER. However, SUM44-LTED cells which continued to express ER showed no specific response to CXCR7 depletion. Furthermore HCC1428-LTED lost expression of both *CXCL11* and *CXCL12*, a feature associated with chemotactic response to ligand gradients and not associated with proliferation [30]. In contrast, MCF7-LTED cells were significantly and specifically inhibited by depletion of CXCR7 either using a siRNA or by chemical intervention with CCX733. This suggests that the increase in *CXCR7* expression and activity may be constrained by the genetic background of MCF7 cells, which remain the most clinically representative cell line modelling ER+ luminal BC.

Contrary to classical chemokine receptors, CXCR7 does not respond to ligand binding by mobilising intracellular calcium via G protein°Coupled receptors, and recent studies have proposed that CXCR7 may be capable of associating and signalling via β-arrestin-mediated pathways [21]. β-arrestins are multifunctional adaptor proteins that facilitate receptor trafficking [31] and can act as bona fide signal transducers [32]. More recently, it has been shown that β-arrestins can translocate to the nucleus and regulate transcriptional events [33], including expression of the antiapoptotic protein BCL2 [34]. However, in our studies, although there was a noticeable decrease in *BCL2* mRNA (discussed below), si*CXCR7* had no effect on BCL2 protein expression or apoptosis and had no effect on β-arrestin expression.

Strikingly, whilst wt-MCF7 and MCF7-LTED cells expressed similar levels of β-arrestin 1, MCF7-LTED cells showed loss of β-arrestin 2. This could have several effects on the ability of the MCF7-LTED cells to proliferate in the absence of E2. First, loss of β-arrestins leads to chromosomal instability, a feature often observed in BC [35],[36], and may provide the LTED cells with a plastic genetic background. Second, a previous study has shown that β-arrestin 2 acts as a corepressor of androgen receptor (AR) suppressing AR-mediated transcription. Given that loss of β-arrestin 2 has been shown to promote AR transcriptional activity, loss of β-arrestin 2 in MCF7-LTED cells might also enhance ER-mediated transcription in the absence of exogenous E2 [37].

Our data suggest that CXCR7 most likely played a role in cell cycle progression in the MCF7-LTED cell line. This was confirmed by the significant reduction in expression of cell cycle proteins cyclin D1, CDK4 and phosphorylated Rb in MCF7-LTED cells, but not in wt-MCF7 cells, in response CXCR7 depletion. Consistent with our data, in recent studies of metastatic prostate cancer cell lines, researchers found that si*CXCR7* caused multiple antitumour effects, including a decrease in proliferation and cell cycle arrest in the G_1_ phase, as well as expression of proteins involved in G_1_/S phase progression [38].

As the MCF7-LTED cells are dependent on ER-mediated transcription for proliferation [5],[39], we hypothesised that CXCR7, via its interaction with the signal transduction kinases, could lead to ligand-independent activation of ER. For instance, previous studies have shown that CXCL12/CXCR4 can lead to activation of downstream signalling pathways implicated in the development of resistance to endocrine therapy, such as mitogen-activated protein kinase family members as well as PI3K/Akt cascades [40],[43].

si*CXCR7* significantly reduced ER-mediated transactivation as measured by an ERE luciferase-linked reporter construct in MCF7-LTED cells, but not in wt-MCF7 cells, in the absence of E2. However, no decrease in expression of kinases associated with ligand-independent or ligand-dependent ER phosphorylation, such p38, pERK1/2, pAKT, pJNK or pCDK7 [44],[45] was evident in response to CXCR7 depletion. Furthermore, the main phosphorylation sites on ER, serine 118 and serine 167, also were not significantly altered.

ER-mediated transcription is also dependent on the recruitment of nuclear coactivators, which are similarly controlled by phosphorylation [46]. One of the major coactivators associated with endocrine resistance is PELP1/MNAR [47]. Previous studies have shown that CDK4-mediated phosphorylation of PELP1 is critical for its oncogenic function and its association with ER [23]. We hypothesised that CXCR7, via crosstalk with PELP1, was leading to enhanced ER-mediated transcription in the absence of E2 in the MCF7-LTED cell line. As expected, si*CXCR7* decreased the association between ER and PELP1 in MCF7-LTED cells and was associated with a concomitant decrease in expression of the endogenous E2-regulated gene *TFF1*. Of interest, *BCL2* is also a classically ER-ERE-mediated gene. We previously noted that mRNA levels were suppressed by si*CXCR7*, providing further support for this mechanism.

## Conclusions

By using an *in vitro* model of endocrine resistance, we have provided evidence of novel crosstalk between ER and CXCR7 that leads to ligand-independent, ER-mediated transactivation via the ER coactivator PELP1. Furthermore, the therapeutic potential of directly targeting this unique crosstalk was highlighted by the antiproliferative effect of the CXCR7 antagonist CCX733. *CXCR7* expression associates clinically with poor overall survival in patients with ER+ BC. Overall, these data indicate that CXCR7 may be both a potential biomarker and a therapeutic target in endocrine-resistant BC that warrants further clinical investigation.

## Authors' contribution

LAM, MD and RR conceived and designed study. RR, SP, AR performed experiments; ZG, QG analysed microarray data sets; AD generated clinical samples; LAM and RR interpreted data and wrote the manuscript. All authors agreed to be accountable for all aspects of the work and ensuring accuracy and integrity and approved the final version of this manuscript.

## Additional files

## Electronic supplementary material

Additional file 1: Figure S1.: Identification of genes associated with resistance to aromatase inhibitor therapy. Intersection of genes from patients treated with neoadjuvant anastrazole that predict for a poor change in Ki67 (*P* <0.01) with genes associated with adaptation of wt-MCF7 cells to LTED (*P* <0.001). (PDF 98 KB)

Additional file 2: Figure S2.: Oestrogen receptor expression analysis in a panel of human breast cancer cell lines and their LTED derivatives. MCF7-LTED, SUM44-LTED and HCC1428-LTED cells keep expression of ER, but T47D-LTED and ZR75-LTED loose ER expression. (PDF 85 KB)

Additional file 3: Figure S3.: Depletion of CXCR4 and CXCR7 causes no additional antiproliferative effect versus si*CXCR7* alone. wt-MCF7, MCF7-LTED, wt-SUM44 and SUM44-LTED cells were transfected with si*CXCR7* alone or with a combination of si*CXCR4* and si*CXCR7*. (PDF 99 KB)

Additional file 4: Figure S4.: Association of CXCR4 and CXCR7 with recurrence in oestrogen receptor-positive breast cancer treated with endocrine therapy and *in vitro* modelling of short (STED) or late oestrogen deprivation (LTED). **(A)** Kaplan-Meier analysis of *CXCR4* and *CXCR7* over 0-5years in ER+ BC patients from a series of 840 patients treated with tamoxifen. Data was stratified by the highest quartile versus the rest. **(B)** Expression of *CXCR4* and *CXCR7* in wt-MCF7 cells and short (STED) or long term oestrogen deprivation (LTED). (PDF 97 KB)

Additional file 5: Figure S5.: Deconvolution of CXCR7 siRNA SMARTpool. MCF7-LTED cells were transfected with each siRNA within the CXCR7 SMARTpool individual. All except oligo 4 resulted in a significant decrease in proliferation. (PDF 42 KB)

Additional file 6: Figure S6.: Protein expression of CXCR7. **(A)** MCF7-LTED cells were transfected with si*control* or si*CXCR7*. After 48-hours monolayers were stained for CXCR7 and visualised by confocal microscopy. **(B)** MCF7-LTED, wt-MCF7 and MDA MB 231 (negative control) cells were formalin fixed and paraffin embedded. Sections were stained for expression of CXCR7. **(C)** Wt-MCF7, MCF7-LTED and MDA MB 261 cells were stained for CXCR7 and expression visualised by FACS. (PDF 11 MB)

Additional file 7: Figure S7.: Assessment of apoptosis. **(A)** MCF7-LTED cells were transfected with si*control* or si*CXCR7*. After 24-hours cells were treated × E2. 24-hours cells later monolayers were stained with PI and the fraction of cells in sub-G_1_ determined by FACS. Data is expressed as percentage relative to si*control*. **(B)** Cells were seeded into 24-well plates, transfected with the siRNAs indicated and 48-hours later assessed for apoptosis using a live/dead assay (Roche Life Science). Data are expressed as fold changes relative to si*control*. (PDF 51 KB)

Additional file 8: Figure S8.: Effect of the combination of si*CXCR7* with fulvestrant (ICI 182,780) on proliferation of MCF7-LTED. MCF7-LTED cells were transfected with si*control* or si*CXCR7* and followed by treatment with 1 nM of fulvestrant in the presence of oestrogen. (PDF 44 KB)

Below are the links to the authors’ original submitted files for images.

Authors’ original file for figure 1

Authors’ original file for figure 2

Authors’ original file for figure 3

Authors’ original file for figure 4

Authors’ original file for figure 5

Authors’ original file for figure 6

## Abbreviations

AI: Aromatase inhibitor
AR: Androgen receptor
BC: Breast cancer
CXCR: C-X-C chemokine receptor
DCC: Dextran-coated charcoal
ER: Oestrogen receptor
LTED: Long-term oestrogen deprivation
PBS: Phosphate-buffered saline

## References

[1] Masamura S, Santner SJ, Heitjan DF, Santen RJ (1995). Estrogen deprivation causes estradiol hypersensitivity in human breast cancer cells. J Clin Endocrinol Metab.

[2] Coutts AS, Murphy LC (1998). Elevated mitogen-activated protein kinase activity in estrogen-nonresponsive human breast cancer cells. Cancer Res.

[3] Santen R, Jeng MH, Wang JP, Song R, Masamura S, McPherson R, Santner S, Yue W, Shim WS (2001). Adaptive hypersensitivity to estradiol: potential mechanism for secondary hormonal responses in breast cancer patients. J Steroid Biochem Mol Biol.

[4] Chan CM, Martin LA, Johnston SR, Ali S, Dowsett M (2002). Molecular changes associated with the acquisition of oestrogen hypersensitivity in MCF-7 breast cancer cells on long-term oestrogen deprivation. J Steroid Biochem Mol Biol.

[5] Martin LA, Farmer I, Johnston SRD, Ali S, Marshall C, Dowsett M (2003). Enhanced estrogen receptor (ER) α, ERBB2, and MAPK signal transduction pathways operate during the adaptation of MCF-7 cells to long term estrogen deprivation. J Biol Chem.

[6] Sabnis GJ, Jelovac D, Long B, Brodie A (2005). The role of growth factor receptor pathways in human breast cancer cells adapted to long-term estrogen deprivation. Cancer Res.

[7] Masri S, Phung S, Wang X, Wu X, Yuan YC, Wagman L, Chen S (2008). Genome-wide analysis of aromatase inhibitor-resistant, tamoxifen-resistant, and long-term estrogen-deprived cells reveals a role for estrogen receptor. Cancer Res.

[8] Martin LA, Ghazoui Z, Weigel MT, Pancholi S, Dunbier A, Johnston S, Dowsett M (2011). An in vitro model showing adaptation to long-term oestrogen deprivation highlights the clinical potential for targeting kinase pathways in combination with aromatase inhibition. Steroids.

[9] Ellis MJ, Tao Y, Young O, White S, Proia AD, Murray J, Renshaw L, Faratian D, Thomas J, Dowsett M, Krause A, Evans DB, Miller WR, Dixon JM (2006). Estrogen-independent proliferation is present in estrogen-receptor HER2-positive primary breast cancer after neoadjuvant letrozole. J Clin Oncol.

[10] Duda DG, Kozin SV, Kirkpatrick ND, Xu L, Fukumura D, Jain RK (2011). CXCL12 (SDF1α)-CXCR4/CXCR7 pathway inhibition: an emerging sensitizer for anticancer therapies?. Clin Cancer Res.

[11] Weigel MT, Ghazoui Z, Dunbier A, Pancholi S, Dowsett M, Martin LA (2012). Preclinical and clinical studies of estrogen deprivation support the PDGF/Abl pathway as a novel therapeutic target for overcoming endocrine resistance in breast cancer. Breast Cancer Res.

[12] Dunbier AK, Ghazoui Z, Anderson H, Salter J, Nerurkar A, Osin P, A’Hern R, Miller WR, Smith IE, Dowsett M (2013). Molecular profiling of aromatase inhibitor-treated postmenopausal breast tumors identifies immune-related correlates of resistance. Clin Cancer Res.

[13] Györffy B, Lanczky A, Eklund AC, Denkert C, Budczies J, Li Q, Szallasi Z (2010). An online survival analysis tool to rapidly assess the effect of 22,277 genes on breast cancer prognosis using microarray data of 1,809 patients. Breast Cancer Res Treat.

[14] Hattermann K, Held-Feindt J, Lucius R, Müerköster SS, Penfold ME, Schall TJ, Mentlein R (2010). The chemokine receptor CXCR7 is highly expressed in human glioma cells and mediates antiapoptotic effects. Cancer Res.

[15] Hartmann TN, Grabovsky V, Pasvolsky R, Shulman Z, Buss EC, Spiegel A, Nagler A, Lapidot T, Thelen M, Alon R (2008). A crosstalk between intracellular CXCR7 and CXCR4 involved in rapid CXCL12-triggered integrin activation but not in chemokine-triggered motility of human T lymphocytes and CD34+ cells. J Leukoc Biol.

[16] Evans AH, Pancholi S, Farmer I, Thornhill A, Evans DB, Johnston SR, Dowsett M, Martin LA (2010). EGFR/HER2 inhibitor AEE788 increases ER-mediated transcription in HER2/ER-positive breast cancer cells but functions synergistically with endocrine therapy. Br J Cancer.

[17] Dowsett M, Nielsen TO, A’Hern R, Bartlett J, Coombes RC, Cuzick J, Ellis M, Henry NL, Hugh JC, Lively T, McShane L, Paik S, Penault-Llorca F, Prudkin L, Regan M, Salter J, Sotiriou C, Smith IE, Viale G, Zujewski JA, Hayes DF (2011). Assessment of Ki67 in breast cancer: recommendations from the International Ki67 in Breast Cancer Working Group. J Natl Cancer Inst.

[18] Levoye A, Balabanian K, Baleux F, Bachelerie F, Lagane B (2009). CXCR7 heterodimerizes with CXCR4 and regulates CXCL12-mediated G protein signaling. Blood.

[19] Hawkins OE, Richmond A (2012). The dynamic yin-yang interaction of CXCR4 and CXCR7 in breast cancer metastasis. Breast Cancer Res.

[20] Berahovich RD, Penfold ME, Schall TJ (2010). Nonspecific CXCR7 antibodies. Immunol Lett.

[21] Rajagopal S, Kim J, Ahn S, Craig S, Lam CM, Gerard NP, Gerard C, Lefkowitz RJ (2010). β-arrestin- but not G protein-mediated signaling by the "decoy" receptor CXCR7. Proc Natl Acad Sci U S A.

[22] Shi Y, Feng Y, Kang J, Liu C, Li Z, Li D, Cao W, Qiu J, Guo Z, Bi E, Zang L, Lu C, Zhang JZ, Pei G (2007). Critical regulation of CD4^+^T cell survival and autoimmunity by β-arrestin 1. Nat Immunol.

[23] Nair BC, Nair SS, Chakravarty D, Challa R, Manavathi B, Yew PR, Kumar R, Tekmal RR, Vadlamudi RK (2010). Cyclin-dependent kinase-mediated phosphorylation plays a critical role in the oncogenic functions of PELP1. Cancer Res.

[24] Burns JM, Summers BC, Wang Y, Melikian A, Berahovich R, Miao Z, Penfold ME, Sunshine MJ, Littman DR, Kuo CJ, Wei K, McMaster BE, Wright K, Howard MC, Schall TJ (2006). A novel chemokine receptor for SDF-1 and I-TAC involved in cell survival, cell adhesion, and tumor development. J Exp Med.

[25] Andre F, Cabioglu N, Assi H, Sabourin JC, Delaloge S, Sahin A, Broglio K, Spano JP, Combadiere C, Bucana C, Soria JC, Cristofanilli M (2006). Expression of chemokine receptors predicts the site of metastatic relapse in patients with axillary node positive primary breast cancer. Ann Oncol.

[26] Su YC, Wu MT, Huang CJ, Hou MF, Yang SF, Chai CY (2006). Expression of CXCR4 is associated with axillary lymph node status in patients with early breast cancer. Breast.

[27] Lapteva N, Yang AG, Sanders DE, Strube RW, Chen SY (2005). CXCR4 knockdown by small interfering RNA abrogates breast tumor growth *in vivo*. Cancer Gene Ther.

[28] Liang Z, Wu H, Reddy S, Zhu A, Wang S, Blevins D, Yoon Y, Zhang Y, Shim H (2007). Blockade of invasion and metastasis of breast cancer cells *via*targeting CXCR4 with an artificial microRNA. Biochem Biophys Res Commun.

[29] Boudot A, Kerdivel G, Habauzit D, Eeckhoute J, Le Dily F, Flouriot G, Samson M, Pakdel F (2011). Differential estrogen-regulation of CXCL12 chemokine receptors, CXCR4 and CXCR7, contributes to the growth effect of estrogens in breast cancer cells. PLoS One.

[30] Luker KE, Lewin SA, Mihalko LA, Schmidt BT, Winkler JS, Coggins NL, Thomas DG, Luker GD (2012). Scavenging of CXCL12 by CXCR7 promotes tumor growth and metastasis of CXCR4-positive breast cancer cells. Oncogene.

[31] Moore CA, Milano SK, Benovic JL (2007). Regulation of receptor trafficking by GRKs and arrestins. Annu Rev Physiol.

[32] DeWire SM, Ahn S, Lefkowitz RJ (2007). Shenoy SK: **β-arrestins and cell signaling**. Annu Rev Physiol.

[33] Kang J, Shi Y, Xiang B, Qu B, Su W, Zhu M, Zhang M, Bao G, Wang F, Zhang X, Yang R, Fan F, Chen X, Pei G, Ma L (2005). A nuclear function of β-arrestin 1 in GPCR signaling: regulation of histone acetylation and gene transcription. Cell.

[34] Frederick TJ, Miller SD (2007). Arresting autoimmunity by blocking β-arrestin 1. Nat Immunol.

[35] Shankar H, Michal A, Kern RC, Kang DS, Gurevich VV, Benovic JL (2010). Non-visual arrestins are constitutively associated with the centrosome and regulate centrosome function. J Biol Chem.

[36] Hara MR, Kovacs JJ, Whalen EJ, Rajagopal S, Strachan RT, Grant W, Towers AJ, Williams B, Lam CM, Xiao K, Shenoy SK, Gregory SG, Ahn S, Duckett DR, Lefkowitz RJ (2011). A stress response pathway regulates DNA damage through β_2_-adrenoreceptors and β-arrestin-1. Nature.

[37] Lakshmikanthan V, Zou L, Kim JI, Michal A, Nie Z, Messias NC, Benovic JL, Daaka Y (2009). Identification of βArrestin2 as a corepressor of androgen receptor signaling in prostate cancer. Proc Natl Acad Sci U S A.

[38] Singh RK, Lokeshwar BL (2011). The IL-8-regulated chemokine receptor CXCR7 stimulates EGFR signaling to promote prostate cancer growth. Cancer Res.

[39] Martin LA, Pancholi S, Chan CM, Farmer I, Kimberley C, Dowsett M, Johnston SR (2005). The anti-oestrogen ICI 182,780, but not tamoxifen, inhibits the growth of MCF-7 breast cancer cells refractory to long-term oestrogen deprivation through down-regulation of oestrogen receptor and IGF signalling. Endocr Relat Cancer.

[40] Zheng H, Dai T, Zhou B, Zhu J, Huang H, Wang M, Fu G (2008). SDF-1α/CXCR4 decreases endothelial progenitor cells apoptosis under serum deprivation by PI3K/Akt/eNOS pathway. Atherosclerosis.

[41] Ghayad SE, Vendrell JA, Ben Larbi S, Dumontet C, Bieche I, Cohen PA (2010). Endocrine resistance associated with activated ErbB system in breast cancer cells is reversed by inhibiting MAPK or PI3K/Akt signaling pathways. Int J Cancer.

[42] Sauvé K, Lepage J, Sanchez M, Heveker N, Tremblay A (2009). Positive feedback activation of estrogen receptors by the CXCL12-CXCR4 pathway. Cancer Res.

[43] Rhodes LV, Short SP, Neel NF, Salvo VA, Zhu Y, Elliott S, Wei Y, Yu D, Sun M, Muir SE, Fonseca JP, Bratton MR, Segar C, Tilghman SL, Sobolik-Delmaire T, Horton LW, Zaja-Milatovic S, Collins-Burow BM, Wadsworth S, Beckman BS, Wood CE, Fuqua SA, Nephew KP, Dent P, Worthylake RA, Curiel TJ, Hung MC, Richmond A, Burow ME (2011). Cytokine receptor CXCR4 mediates estrogen-independent tumorigenesis, metastasis, and resistance to endocrine therapy in human breast cancer. Cancer Res.

[44] Ali S, Coombes RC (2002). Endocrine-responsive breast cancer and strategies for combating resistance. Nat Rev Cancer.

[45] Musgrove EA, Sutherland RL (2009). Biological determinants of endocrine resistance in breast cancer. Nat Rev Cancer.

[46] Xu J, Wu RC, O’Malley BW (2009). Normal and cancer-related functions of the p160 steroid receptor co-activator (SRC) family. Nat Rev Cancer.

[47] Gururaj AE, Rayala SK, Vadlamudi RK, Kumar R (2006). Novel mechanisms of resistance to endocrine therapy: genomic and nongenomic considerations. Clin Cancer Res.

