# Supplementary figures and images for: Identification of chemokine receptors as potential modulators of endocrine resistance in oestrogen receptor–positive breast cancers
Source: Breast Cancer Res. 2014 Oct 31;16:447. doi: 10.1186/s13058-014-0447-1 (PMC4303127; doi:10.1186/s13058-014-0447-1)

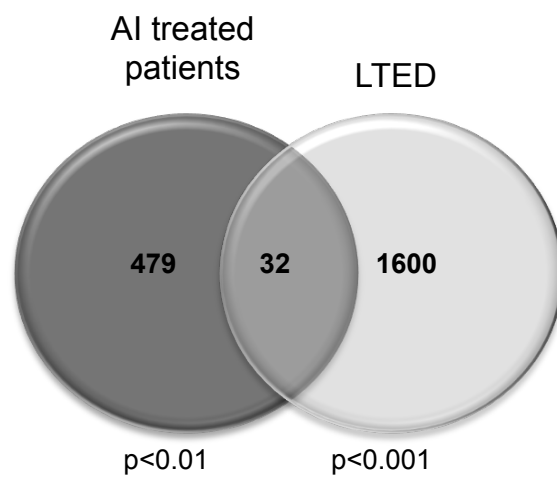

Supplement: Supplementary file 1 — Additional file 1: Figure S1.: Identification of genes associated with resistance to aromatase inhibitor therapy. Intersection of genes from patients treated with neoadjuvant anastrazole that predict for a poor change in Ki67 (P <0.01) with genes associated with adaptation of wt-MCF7 cells to LTED (P <0.001). (PDF 98 KB) [file 13058_2014_447_MOESM1_ESM.pdf]

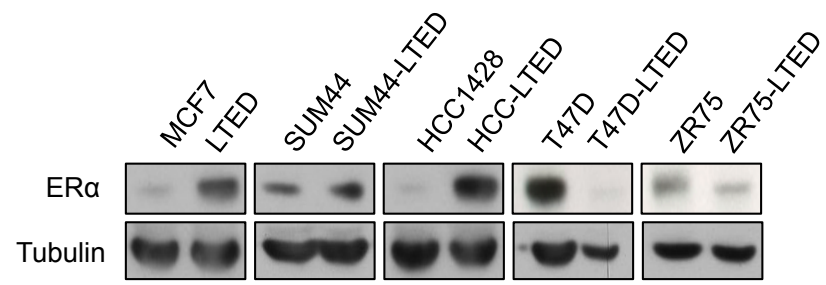

Supplement: Supplementary file 2 — Additional file 2: Figure S2.: Oestrogen receptor expression analysis in a panel of human breast cancer cell lines and their LTED derivatives. MCF7-LTED, SUM44-LTED and HCC1428-LTED cells keep expression of ER, but T47D-LTED and ZR75-LTED loose ER expression. (PDF 85 KB) [file 13058_2014_447_MOESM2_ESM.pdf]

**wt-MCF7**

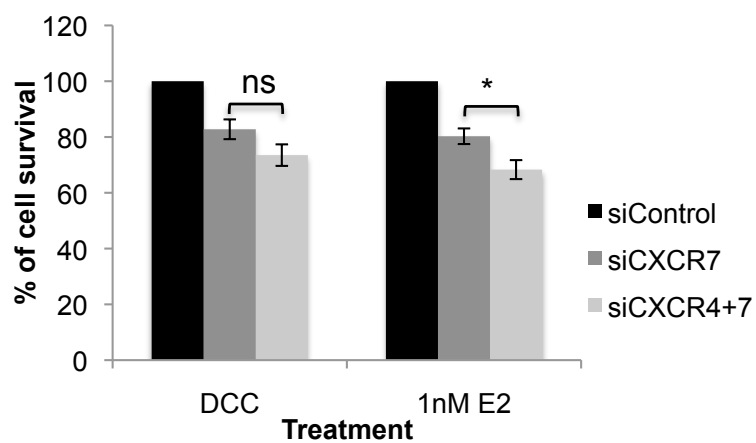

**MCF7-LTED**

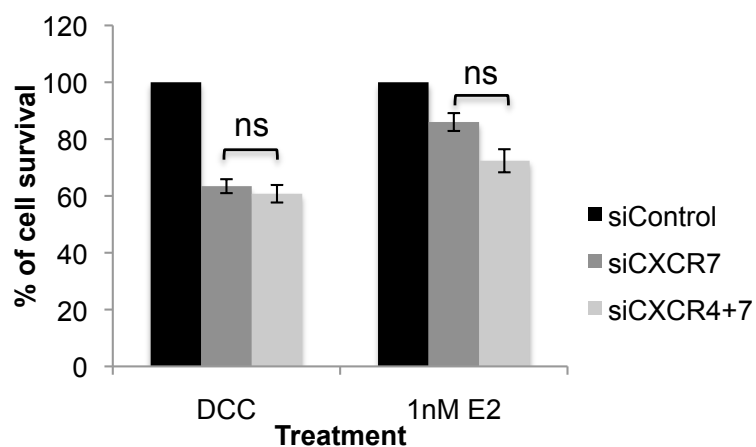

**wt-SUM44**

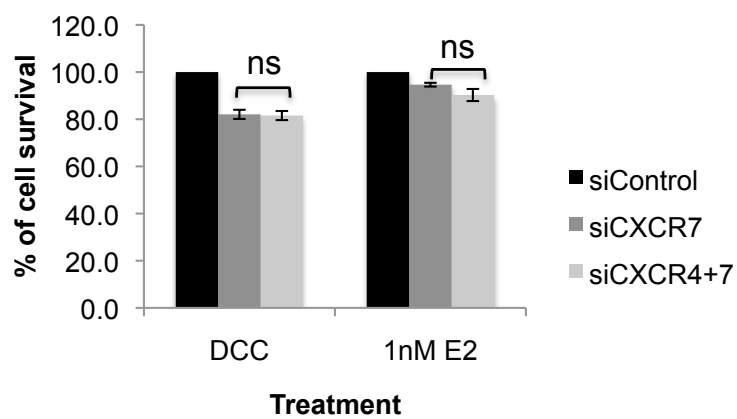

**SUM44-LTED**

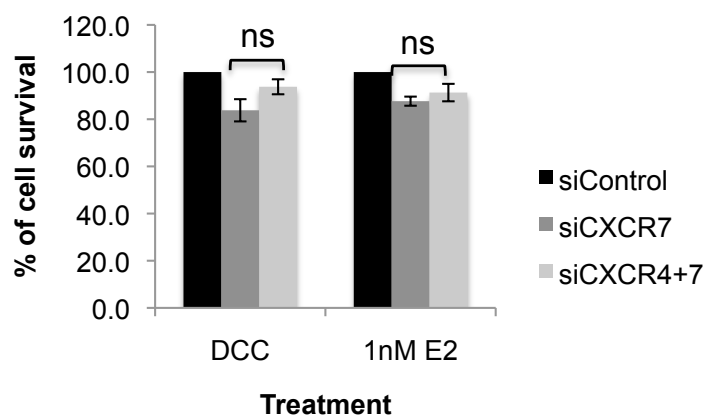

Supplement: Supplementary file 3 — Additional file 3: Figure S3.: Depletion of CXCR4 and CXCR7 causes no additional antiproliferative effect versus siCXCR7 alone. wt-MCF7, MCF7-LTED, wt-SUM44 and SUM44-LTED cells were transfected with siCXCR7 alone or with a combination of siCXCR4 and siCXCR7. (PDF 99 KB) [file 13058_2014_447_MOESM3_ESM.pdf]

**A**

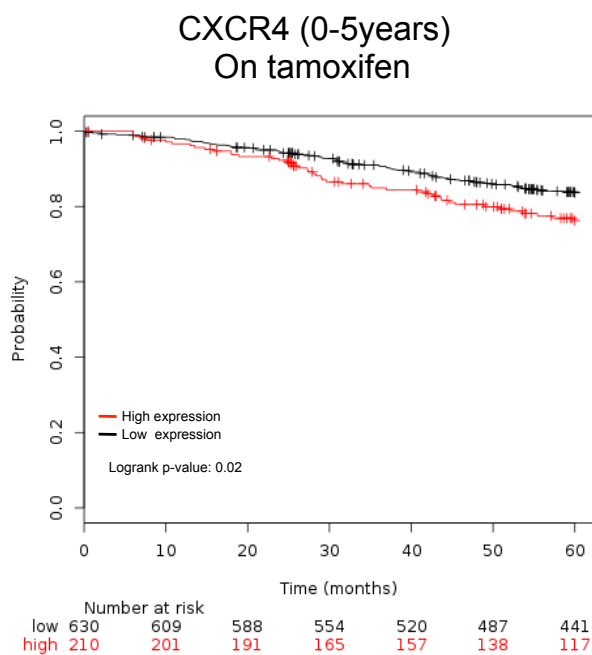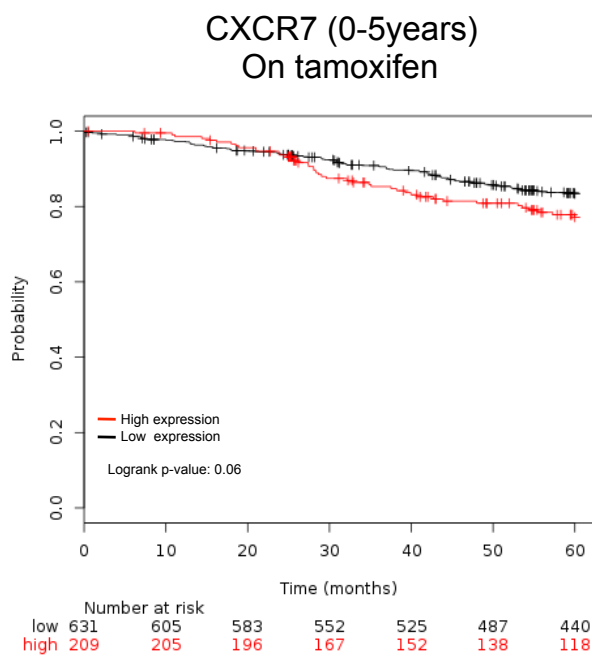

**B**

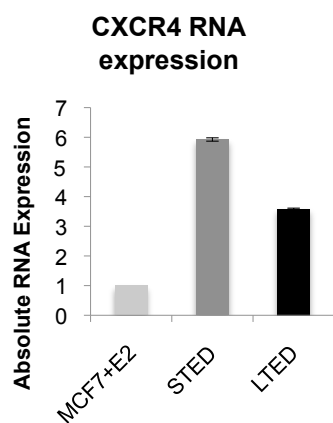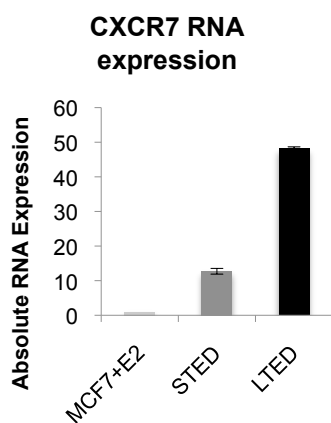

Supplement: Supplementary file 4 — Additional file 4: Figure S4.: Association of CXCR4 and CXCR7 with recurrence in oestrogen receptor-positive breast cancer treated with endocrine therapy and in vitro modelling of short (STED) or late oestrogen deprivation (LTED). (A) Kaplan-Meier analysis of CXCR4 and CXCR7 over 0-5years in ER+ BC patients from a series of 840 patients treated with tamoxifen. Data was stratified by the highest quartile versus the rest. (B) Expression of CXCR4 and CXCR7 in wt-MCF7 cells and short (STED) or long term oestrogen deprivation (LTED). (PDF 97 KB) [file 13058_2014_447_MOESM4_ESM.pdf]

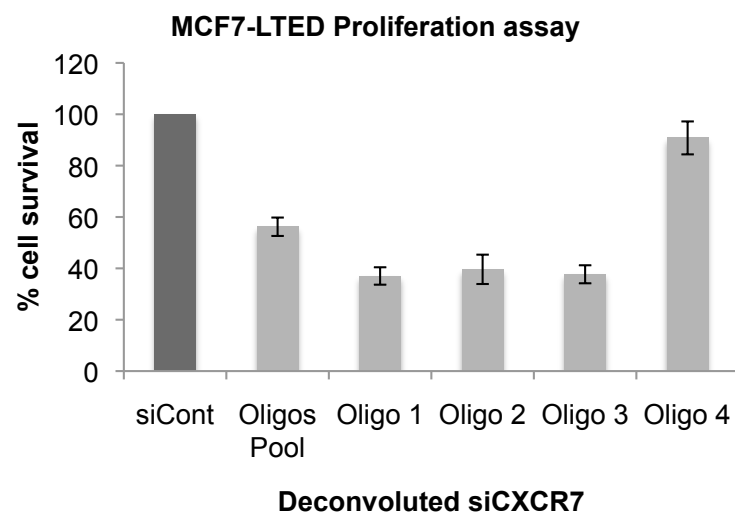

Supplement: Supplementary file 5 — Additional file 5: Figure S5.: Deconvolution of CXCR7 siRNA SMARTpool. MCF7-LTED cells were transfected with each siRNA within the CXCR7 SMARTpool individual. All except oligo 4 resulted in a significant decrease in proliferation. (PDF 42 KB) [file 13058_2014_447_MOESM5_ESM.pdf]

A

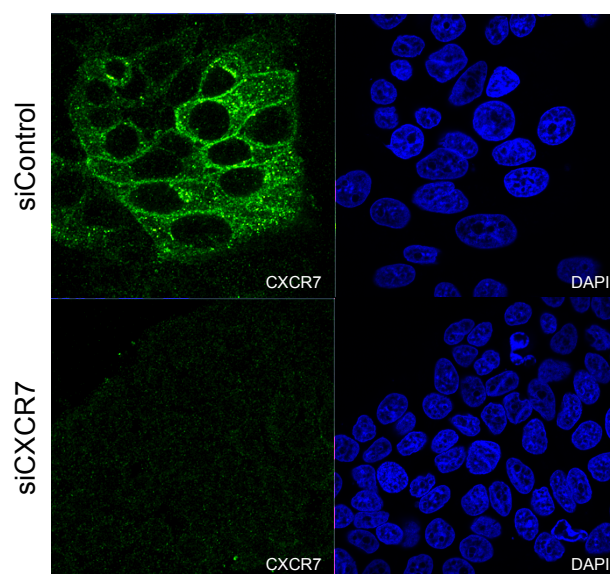

B

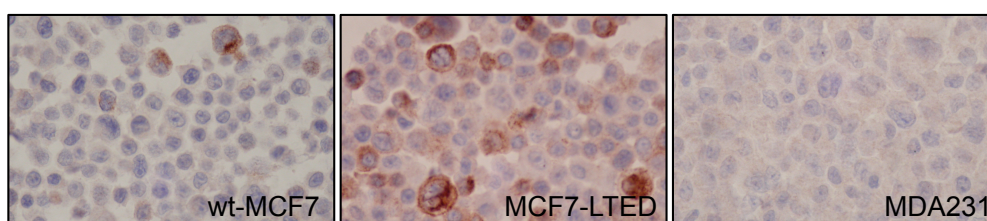

C

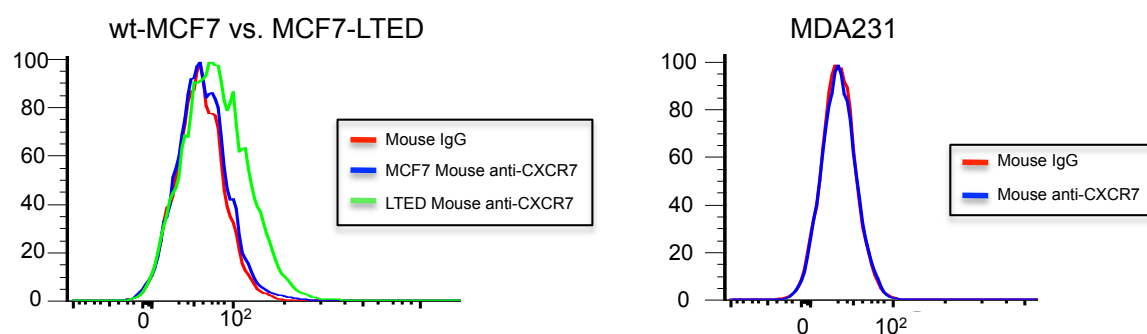

Supplement: Supplementary file 6 — Additional file 6: Figure S6.: Protein expression of CXCR7. (A) MCF7-LTED cells were transfected with sicontrol or siCXCR7. After 48-hours monolayers were stained for CXCR7 and visualised by confocal microscopy. (B) MCF7-LTED, wt-MCF7 and MDA MB 231 (negative control) cells were formalin fixed and paraffin embedded. Sections were stained for expression of CXCR7. (C) Wt-MCF7, MCF7-LTED and MDA MB 261 cells were stained for CXCR7 and expression visualised by FACS. (PDF 11 MB) [file 13058_2014_447_MOESM6_ESM.pdf]

**A**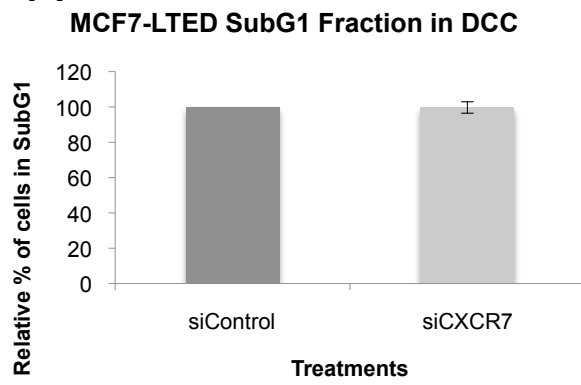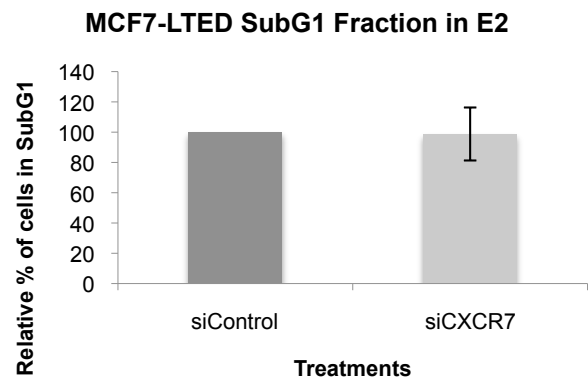**B**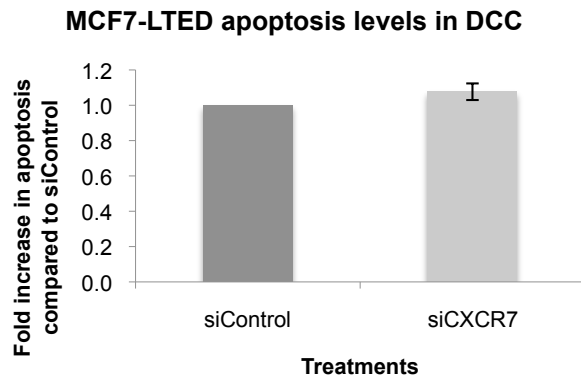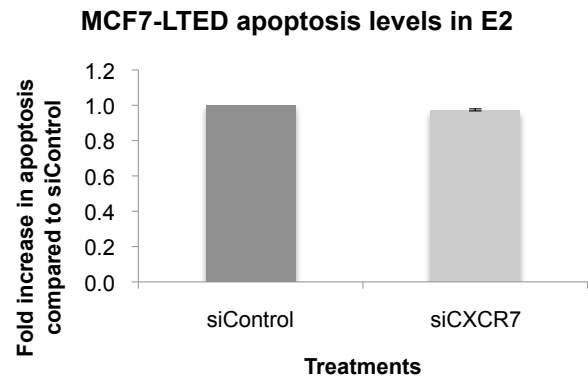

Supplement: Supplementary file 7 — Additional file 7: Figure S7.: Assessment of apoptosis. (A) MCF7-LTED cells were transfected with sicontrol or siCXCR7. After 24-hours cells were treated × E2. 24-hours cells later monolayers were stained with PI and the fraction of cells in sub-G1 determined by FACS. Data is expressed as percentage relative to sicontrol. (B) Cells were seeded into 24-well plates, transfected with the siRNAs indicated and 48-hours later assessed for apoptosis using a live/dead assay (Roche Life Science). Data are expressed as fold changes relative to sicontrol. (PDF 51 KB) [file 13058_2014_447_MOESM7_ESM.pdf]

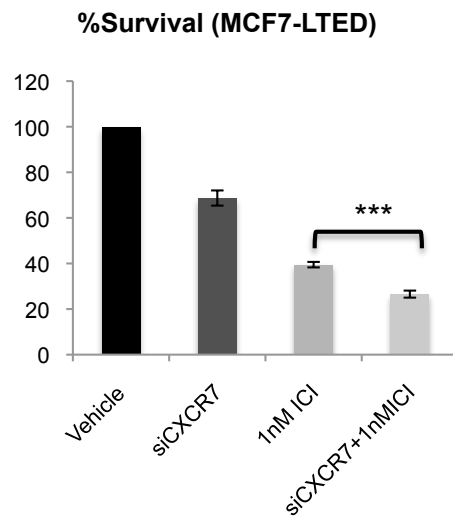

Supplement: Supplementary file 8 — Additional file 8: Figure S8.: Effect of the combination of siCXCR7 with fulvestrant (ICI 182,780) on proliferation of MCF7-LTED. MCF7-LTED cells were transfected with sicontrol or siCXCR7 and followed by treatment with 1 nM of fulvestrant in the presence of oestrogen. (PDF 44 KB) [file 13058_2014_447_MOESM8_ESM.pdf]

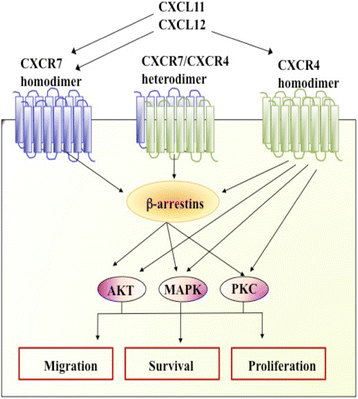

Supplement: Supplementary file 9 — Authors’ original file for figure 1 [file 13058_2014_447_MOESM9_ESM.gif]

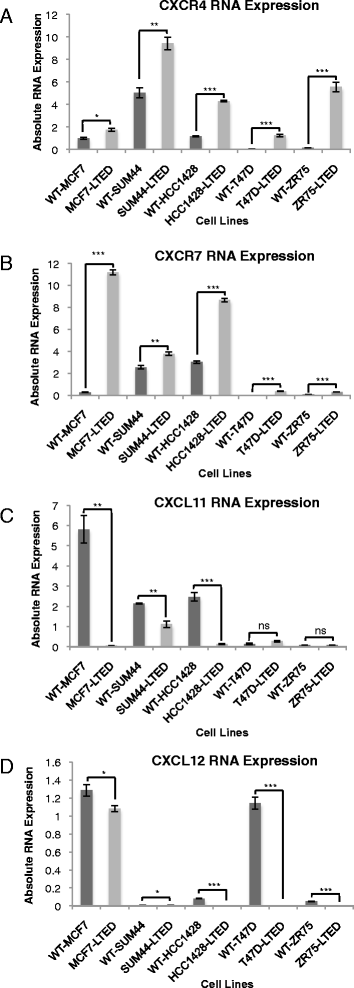

Supplement: Supplementary file 10 — Authors’ original file for figure 2 [file 13058_2014_447_MOESM10_ESM.gif]

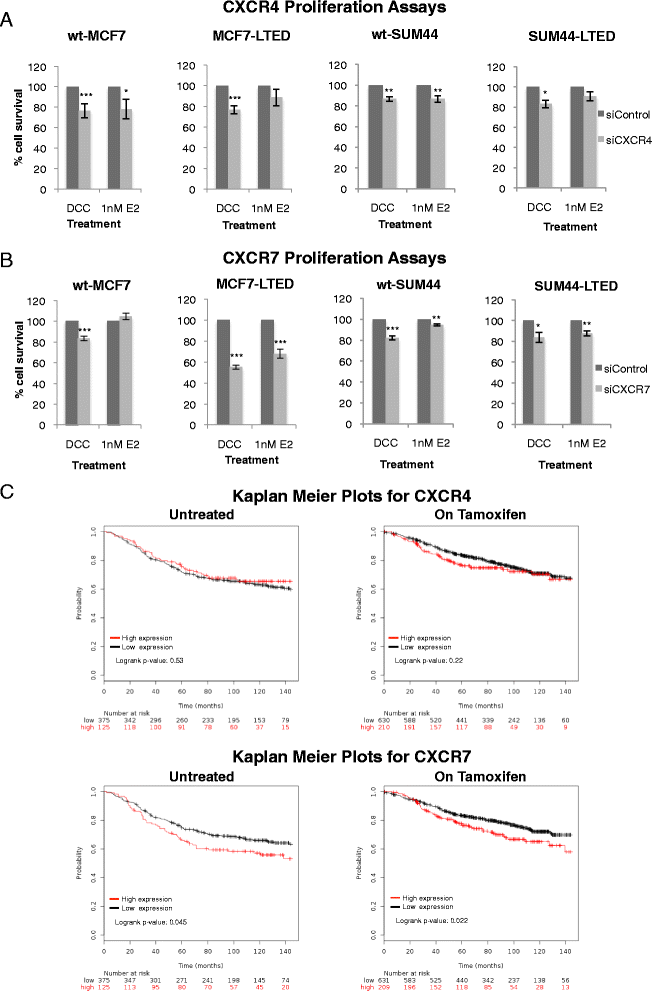

Supplement: Supplementary file 11 — Authors’ original file for figure 3 [file 13058_2014_447_MOESM11_ESM.gif]

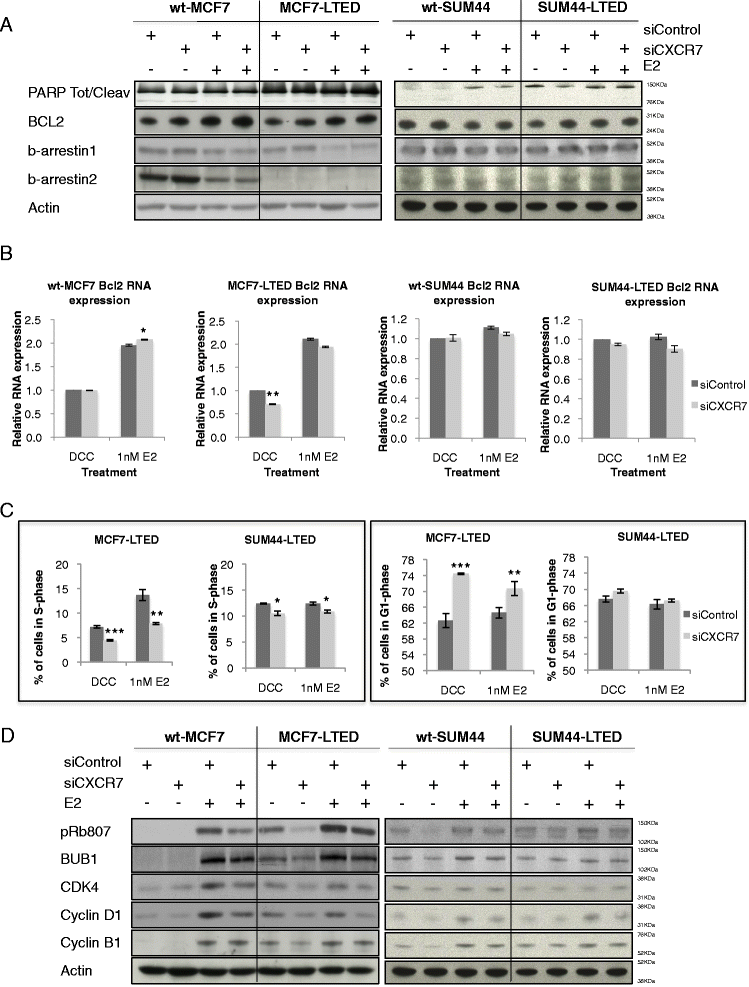

Supplement: Supplementary file 12 — Authors’ original file for figure 4 [file 13058_2014_447_MOESM12_ESM.gif]

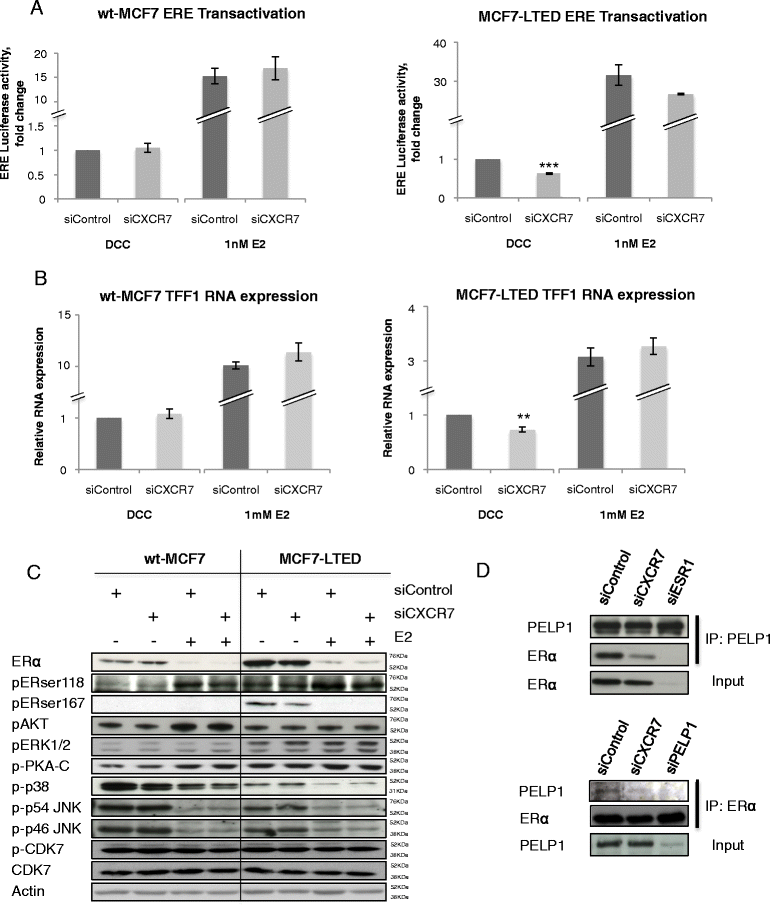

Supplement: Supplementary file 13 — Authors’ original file for figure 5 [file 13058_2014_447_MOESM13_ESM.gif]

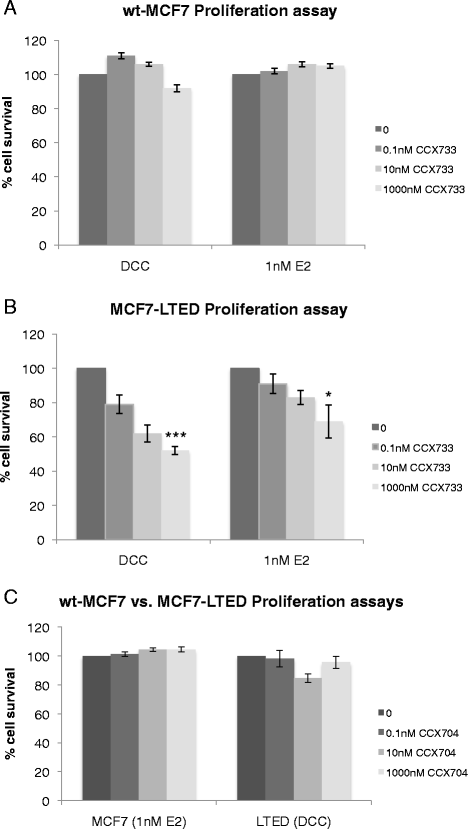

Supplement: Supplementary file 14 — Authors’ original file for figure 6 [file 13058_2014_447_MOESM14_ESM.gif]
